# Supplementary material for: Cattle-related occupational accidents in Japan
Source: PLoS One. 2023 Jul 28;18(7):e0289210. doi: 10.1371/journal.pone.0289210 (PMC10381074; doi:10.1371/journal.pone.0289210)
Supplement: Attachment — Submitted filename: Response to Reviewers.docx [file pone.0289210.s002.docx]

Response to Reviewers

Dr Pramod K Pandey

Academic Editor

PLOS ONE

Sep 12, 2022

Dear Dr Pramod K Pandey

Thank you for your ongoing consideration of our manuscript for publication in PLOS ONE. We appreciate the time spent by you and the reviewers and believe the revised manuscript is improved. Below, we have addressed your and the reviewers’ comments.

We look forward to your editorial decision.

Sincerely,

Hilomi IWAI, MPH

Teikyo University, Faculty of Pharma-Science, Laboratory of Environmental Health and Global Health (on behalf of all the authors)

**EDITOR’S SPECIFIC COMMENTS:**

1. Please ensure that your manuscript meets PLOS ONE's style requirements, including those for file naming. …

[Response]

We ensure that our manuscript meets PLOS ONE's style requirements again.

2. We suggest you thoroughly copyedit your manuscript for language usage, spelling, and grammar. If you do not know anyone who can help you do this, you may wish to consider employing a professional scientific editing service. …

[Response]

Thank you for your kindly advise. Our manuscript had already been reviewed by the reviser before the submission. The citification will be submitted with this letter.

3. Thank you for stating the following financial disclosure:

"No. The funders had no role in study design, data collection and analysis, decision to publish, or preparation of the manuscript."…

[Response]

“The authors received no specific funding for this work.”

4. PLOS requires an ORCID iD for the corresponding author in Editorial Manager on papers submitted after December 6th, 2016. …

[Response]

Our ORCID iD are

Hilomi IWAI:0000-0003-2884-3651

Hideki YAMAMOTO: 0000-0003-0127-759X

5. Please include a separate caption for each figure in your manuscript.

[Response]

We submitted the each figure in our manuscript in each file.

**REVIWER’S SPECIFIC COMMENTS:**

Reviewer #1: The manuscript present a EDA of the cattle involved occupational accidents related to and provides an insight on health and safety issues in cattle production through claim data between 2013 to 2016. However, having access to a large dataset could be used to find more complex modelling and providing a better picture in future.

[Response]

We thank the reviewer for the positive comment. Gathering the information of agricultural occupational accidents is under consideration issues. In Japan, the most of agriculture-related accidents are not included in the Survey on Industrial Accidents (by Ministry of Health, Labour and Welfare of the Japanese government) because 98% of Japan's agricultural management entities are family-owned (self-employed) and have no compulsory occupational accident insurance or obligation to report. We expect the positive approach by the related government ministries and agencies.

Reviewer #2 Dr Mahmoud M. Nour: All comments are attached in the PDF which I included all my corrections and feedback for the authors regarding the manuscript titled cattle-related injuries and fatalities in Japan. Great work if you consider all comments from all reviewers.

[Response]

Thank you very much for providing important comments. We are appreciate for the time and energy you expended. We revised as follows.

Line17 etc. (Line19 etc. in the new file): ‘Incident’ or ‘Accident’

We revised the ward according to your advice. We used ‘Accident’, because ‘Accident’ is cases which results in fatal or non-fatal injury and ‘Incident’ is cases witch results in non-human injury: analysed JA Kyosai mutual aid payment claim form data include only the treated in hospital cases. However, event is ‘incident’ in Epidemiology as your advice. Therefore, we revised ‘Accident’ to ‘Incident’.

Line56, 178, 179(Line73 to 89, 259 to 263 in the new file): zoos

We revised the text to reflect according to your advice as follow. We wanted to explain that cattle are kind of large herbivores need to be kept with caution. But the sentences of the first edition were little bet difficult to make sense.

Line56(Line73 to 89)

Before: ‘Although cattle, a kind of large herbivore, might not look dangerous, the Ministry of the Environment of the Japanese government recommends keeping them under protected contact in zoos.’

After: ‘Even if cattle look gentle, they are a kind of large herbivore. Zoos that also deal with animals are required to keep large herbivore under protected contact as dangerous animals by the Ministry of the Environment of the Japanese government having jurisdiction over zoos.’

Line178,179(Line259 to 263 in the new file)

Before: ‘	Cattle are common farm animals; under protected contact keeping is recommended in zoos by the Ministry of the Environment of the Japanese government. Not only in zoos but also in farms, cattle should be kept under protected contact. Even farmers with long and varied experience with cattle cannot predict their behaviour. Preventions have limits and harm reduction must be considered on the other hand.’

After: ‘Beef and milk cattle are common farm animals. However, they are large herbivorous need to keep with caution. Even farmers with significant experience with cattle cannot predict their behaviour. Measures of harm reduction in incident should be taken at the same time as measures of prevention.’

Line127(Line 187 in the new file): ‘Bringing’ or ‘Handling’

We would like to keep ‘Bringing’. Because ‘bringing’ of ‘Activity at the moment of incident’ means ‘let cattle move from some place to another place’. And ‘Handling’ sometimes means ‘general breeding work’. Therefor we chose ‘bringing’ according to the previous study (David I. Douphrate et.al. Livestock-Handling Injuries in Agriculture: An Analysis of Colorado Workers’ Compensation Data. AMERICAN JOURNAL OF INDUSTRIAL MEDICI, 2009;52:391-407.).

Other pointed wards and sentences

We revised according to your advice.
