# Supplementary material for: Cattle-related occupational accidents in Japan
Source: PLoS One. 2023 Jul 28;18(7):e0289210. doi: 10.1371/journal.pone.0289210 (PMC10381074; doi:10.1371/journal.pone.0289210)
Supplement: Attachment — Submitted filename: Response to Reviewers.docx [file pone.0289210.s004.docx]

Response to Reviewers

Dr Pramod K Pandey

Academic Editor

PLOS ONE

Mar 13, 2023

Dear Dr Pramod K Pandey

Thank you for your ongoing consideration of our manuscript for publication in PLOS ONE. We appreciate the time spent by you and the reviewers and believe the revised manuscript is improved. Below, we have addressed your and the reviewers’ comments.

We look forward to your editorial decision.

Sincerely,

Hilomi IWAI, MPH

Teikyo University, Faculty of Pharma-Science, Laboratory of Environmental Health and Global Health (on behalf of all the authors)

**EDITOR’S SPECIFIC COMMENTS:**

1. Please review your reference list to ensure that it is complete and correct. …

[Response]

We reviewed our reference list, and ensured all reference were correct. However, sometimes they may not directly searchable because they are originally written by Japanese. If it is necessary, list written by Japanese can be added.

**REVIWER’S SPECIFIC COMMENTS:**

Reviewer #1 Dr Masoud Shirali:

We thank Dr Masoud Shirali for the positive comment.

Reviewer #2 Dr Mahmoud M. Nour:

[Response]

Thank you very much for providing important comments. We are appreciate for the time and energy you expended. We revised as follows.

Line1 (Line1in the new file):

After careful thought, we would like to change title from ‘Cattle-related injuries in Japan’ to ‘Cattle-related Occupational Accidents in Japan’. According to your kind advice, we made it more accountable about the study.

Line23 (Line27 in the new file):

We would like to keep ‘them’ to describe more clearly. This ‘them’ means ‘extracted data set’ and it is different from the original data.

Line24 (Line28 in the new file):

We would like to keep original sentence. Because we describe age as age group and not average age.

Line124 etc. (Line148 etc. in the new file):

We would like to keep original sentence. We would describe more pointed span and it is a set idiom.

Other pointed words and sentences:

We revised according to your advice.
