# Supplementary material for: Cattle-related occupational accidents in Japan
Source: PLoS One. 2023 Jul 28;18(7):e0289210. doi: 10.1371/journal.pone.0289210 (PMC10381074; doi:10.1371/journal.pone.0289210)
Supplement: Attachment — Submitted filename: Response to Reviewers.docx [file pone.0289210.s005.docx]

Response to Reviewers

Dr Pramod K Pandey

Academic Editor

PLOS ONE

Jun 23, 2023

Dear Dr Pramod K Pandey

Thank you for your ongoing consideration of our manuscript for publication in PLOS ONE. We do appreciate the time spent by you and the reviewers and believe the revised manuscript is improved. Below, we have addressed your and the reviewers’ comments.

We look forward to your editorial decision.

Sincerely,

Hilomi IWAI, MPH

Teikyo University, Faculty of Pharma-Science, Laboratory of Environmental Health and Global Health (on behalf of all the authors)

**REVIWER’S SPECIFIC COMMENTS:**

Reviewer #1: Dr Masoud Shirali

[Response]

Thank you very much for providing important comments. We appreciate for the time and energy you expended.

Reviewer #3:

[Response]

Thank you very much for providing important comments. We appreciate for the time and energy you expended. We revised as follows.

1. The number of populations working for agriculture: Though, we didn’t write about it to keep our article simple, according to your advice, we added the number of populations working for agriculture (national population census of Japan in 2015). However, there are concerns that the number dose not fully reflect the actual situation. Because there so many part-time worker and family helper working in farm in busy farming season. And those people are not counted for population mainly working for agriculture. (Line 54~58)
2. The number of policyholders: Though, we didn’t write about it to keep our article simple, and JA Kyosai does not disclose the number of policyholders for individual insurance products, according to your advice, we added the released number of policyholders of comprehensive life insurance. We do not consider it important because our research dose not aim to calculate incident rate or other epidemiological indexes. (Line 89~93)
3. Additional consideration in discussion: We thank you for your deep interest in our work and your empathic advice. We carefully discussed your 2 points of suggestion. First your suggestion is about 3 fatal cases; the reference is the data of Survey on Industrial Incidents, of the Ministry of Health, Labour and Welfare of the Japanese government released by Japan Industrial Safety and Health Association. The data is from another survey; it is difficult to compare to our study directly. And our study discusses only injured case. Second your suggestion is about the report from JA Kyosai report in data of 2017 to 2020; This data represents a newer time period for the data we have been provided with and analysed. These data also need to be considered, but they do not influence the results of our study.　And as a consequence, we have decided to refrain from mentioning these subjects.
4. Thank you for your advice. We agree that ‘Milk cattle’ is more common as spoken. However, we would like to keep ‘Dairy cattle’ because it is more formal and academic.
5. Thank you for correcting our miss typing. We correct it to ‘Ministry of Agriculture, Forestry and Fisheries’ and ‘results’.
